# A Public Bacteriological Laboratory

**Published:** 1898-09-24

**Authors:** 


### The Hospital

A Journal of

The Medical Sciences and Hospital Administration.

Vol. XXIV.—No. 626.] Edited by Sir Henry Burdett, K.C.B., and Solomon C. Smith, M.D., M.R.C.P. [Sept. 24, 1898.

### A Public Bacteriological Laboratory.

It is set forth, in a sort of semi-official communication which appeared last week in several newspapers, that the London County Council has addressed an inquiry to the vestries and district boards of the metropolis, asking them whether they would be in favour of an endeavour on the part of the Council to obtain Parliamentary powers for the establishment of a bacteriological laboratory, the chief purpose of which would be to examine, at the cost of the county, any material derived from suspected cases of infectious disease, and submitted for the purpose either by medical officers of health or by private practitioners, with a view to the establishment of a complete and satisfactory diagnosis. It is said that the vestries of Shoreditch and of St. Pancras have already replied in a sense favourable to the project, and that the Council is likely to receive answers from other bodies in the course of a month or six weeks, or as soon after the vacations as time has been afforded for the full consideration of what is, without doubt, a question of great practical importance. If we take the single case of suspected diphtheria, especially in the not uncommon form of an apparently trivial sore throat in a child attending school, it is manifest that the prompt discovery of the truth might often be the means of preventing a wide diffusion of disease, and it is equally manifest that private medical practitioners, in the great majority of instances, cannot possibly undertake the necessary investigation. Very few of them possess the necessary appliances, and fewer still the time for employing them. A laboratory, to which suspected material might be sent at any time, and in which its character would be promptly reported upon at the public expense, would take away every vestige of an excuse for failing to recognise the nature of the cases referred to; and, especially in dealing with the children of the actually or relatively poor, would in many instances be likely to be of great public advantage.

While so much as this is obvious, it must not be forgotten that the proposed method of attaining the desired result may not be the best which could be suggested. The London County Council is an eminently, it may almost be said a typically, unscientific body; and does not at first sight appear to be well constituted for the control of a purely scientific establishment. It is not very long since the Council, or its Asylums Committee, laid it down, as a condition of the appointment, that the pathologist to its lunatic asylums should not undertake experimentation upon living animals; and a similar prohibition, which it is certain that at least some of the members would desire to impose, would seriously cripple the proceedings of a bacteriologist, and would condemn him to abstain from an important class of work calculated to advance the science in the pursuit of which he was engaged. He would be cut off from original research with regard to the effects produced by pathogenic micro-organisms, and would be compelled to take these effects upon trust from fellow-workers who were more favourably situated. The first result of this would be that bacteriologists of the first class would not accept office under such conditions, and that the control of the proposed institution would have to be committed to inferior men. Even if the "antis" on the Council were unable to carry matters so far as this, they would certainly be able to make themselves hindrances to the proper working of the laboratory, and would in some degree diminish its efficiency and the confidence with which its results would be regarded by the medical profession, and hence, indirectly, by the public.

If the London County Council proposes to establish a bacteriological laboratory for its own purposes, for the investigation of water and sewage, the air of lodging-houses and of workmen's dwellings, and for the hundred and one things that now come in the way of its daily work, that is a more or less domestic arrangement, and we have nothing more to say about it. But if it is proposed to establish a great central public health laboratory for the use of London, we say that the propriety of such a proceeding depends entirely upon the answer which can be given to the question whether such a laboratory will do better work and command greater confidence than such laboratories as could be at once called into existence without asking for any special powers.

We are quite prepared to admit that the decisions arrived at by official examiners, who should be the cream of the profession, working in perfectly fitted laboratories, with all modern appliances and methods at their beck and call, ought to command a respect and meet with a general acceptance which would never be accorded to private investigations conducted in establishments run on either philanthropic or commercial principles. But would a laboratory under the direct control of the London County Council be conducted on purely scientific lines; and would the officials who work therein be allowed to have recourse to all modern methods and appliances? There is the rub; for it must be remembered that, with a great central official laboratory once established, all chance for private enterprise will be gone.

In the present scientific advisers of the County Council we have every confidence, and members of committee, who see the actual work, generally give a loyal support to their permanent officials. But the Council itself stands in a different position; it is swayed by many influences the reverse of scientific, and faddists possess a power therein which tends to lessen its fitness for assuming the control of a work which, if well performed, must tread on the corns of many hypersensitive people.

